# Supplementary material for: "I think we did the best that we could in the space:” A qualitative study exploring individuals’ experiences with three unconventional environments for patients with a delayed hospital discharge
Source: PLoS One. 2024 Feb 27;19(2):e0297542. doi: 10.1371/journal.pone.0297542 (PMC10898730; doi:10.1371/journal.pone.0297542)
Supplement: Attachment — Submitted filename: Unconventional space review.docx [file pone.0297542.s001.docx]

**Long Title:** "I think we did the best that we could in the space: "A qualitative study exploring individuals' experiences with three unconventional environments for patients with a delayed hospital discharge

Overall comment

The authors demonstrated good effort in the conduct of this study by using a combination of observation and interview techniques to confirm the findings. However, the writing skills needed improvement, especially in the flow and coherency. The skill in the presentation of the results needed improvement as well. The current results are considered ''first draft''. More literature search is needed to improve the ''theme'' and related result presentations.

Introduction:

The introduction did not provide an in-depth and clear perspective that the unconventional environments were only referring to pandemic periods. Secondly, the flow of the introduction could be further improved. Thirdly, the authors need to describe the context of delayed discharge. The readers in other countries may have different interpretations of the meaning of delayed discharge. Also, the structure and under what conditions the delayed discharge patients need to be admitted to unconventional environments.

The first paragraph gave the reader an impression that the context of the manuscript was focusing on general perspectives of delayed discharge. The context of delaying hospital discharge should focus on the pandemic period in the first paragraph. For example, a sentence in the third paragraph, ''Patients experiencing a delayed care transition, also known as a delayed discharge or alternate level of care, are a population that was frequently transitioned to unconventional environments during the pandemic,'' should be brought forward to the first paragraph.

Objective

The objectives were unclear; the methods were mainly focused on the experiences during the pandemic, but it was not specified in the objectives per se. Please improve.

''describe the characteristics of three unconventional environments'': there were no prior sentences mentioning three unconventional environments. It is, therefore, uncertain which three unconventional environments were referring to.

Methods

'*'Unit level observations*'':

- How was observation being performed at site C?
- Were the patients or their caregivers at the sites aware of the presence of the study team members during the conduct of the observations?
- Video conferencing: were the videos being turned on during the interview?

*Interview*

- Were the providers and clinical managers recruited being paired?
- Were the patients and caregivers recruited being paired?
- ''participant experiences with receiving or providing care'': the author may need to include the topics, dimensions, or examples of questions covered in the interview.

Data analysis

Although it was being cited, it would be better to include more information about ''leading practices guide for delayed discharge'' including providing the Title of the guide.

''model'': The term ''model'' appeared abruptly in the data analysis without prior introduction. Please improve. The term model of care'' should be brought up in the introduction. Lack of coherency when ''model'' appeared abruptly in the data analysis section.

''In combination with publicly available documents'': what do the publicly available documents refer to? The author may want to clarify this.

''Observations were made during rounds, meetings, huddles, and overall day-to-day functioning.'' this sentence should be included in the method of data collection.

''Further codes were used in order to inform the descriptions outlined in Table 1 of the results: context of the model of care, physical space, staff composition, patient eligibility, and participant role. The information gathered from these codes helped to inform the descriptions outlined in Table 1 of the results.''

**Comment:** The flow was disrupted. It appeared unclear to the reviewer with regard to what ''descriptions'' were outlined in Table 1. What does ''further codes' refer to? The reviewer cannot relate the meaning of further and the description outline in Table 1. It wasn't very clear.

Normally, when mentioning Table 1 in the text, ''Table 1'' should be followed at the end of this paragraph. The author may need to improve the flow.

Saturation points: how were the saturation points being determined?

Credibility and trustworthiness: please describe how these two aspects are being ensured in the process of data analysis.

Results

Overall comments about results: The descriptions are fair; they can be improved in order to make the overall presentation succinct. The definition or description of the meaning of each theme that is derived from the ''leading practice guide'' can be added at the beginning of each theme description.

''A total of 29 individuals participated in this study from the three unconventional

environments.

Comment: please improve this sentence.

Table 1

- The physical environment column can be improved. The current presentation is disorganized and messy.
- Site A: ''Older hospital, renovated in 2018 to address capacity pressures.'' This information did not help the reader to understand the context of A in a better picture.

Table 2

- The interview notes/ summary column needed improvement (more data ''cleaning'') for concise information.
- Staffing model: what is the ''staff model''?
- Frequent, face-to-face team huddles (by floor): what does this mean?

Theme: Implications on staffing models and continuity of care

- Comment: kindly address the definition of ''staffing model'' at the beginning paragraph of this theme.
- What is the meaning of the ''shared staffing model.''?

The last paragraph of the third theme: suggest adding a quote for the description of the last paragraph.

Discussion:

- What do you mean by ''two diverse health regions''?
- Page 26-27, lines 465-472: The author may want to revise this paragraph. The ''healing'' context is important, but the description lacks linkage to the context of the unconventional environment; the authors only linked ''healing'' with delayed discharge.
- ''One component of the framework focuses on the interpersonal environment, including personal and professional relationships.'' Which framework was the author referring to?
- ''Another component of the framework focuses on the behavioural environment, which entails actions taken by oneself or others to promote healing.'' Again, which framework was it referring to?
- The prior sentence was followed by ''Integrative care''. There was a lack of coherency between the prior sentence and ''integrative care''. The audience cannot relate to the connectivity between these two sentences.

Conclusion

''We used constructs from the OHE framework and MMSF to provide insights…'' This OHE framework and MMSF appeared abruptly in the conclusion without prior introduction of these two frameworks in the prior sections. This renders it difficult for the reader to relate the frame to study findings and conclusions.
